# Molecular characterization and genotyping of hepatitis C virus from Sudanese end-stage renal disease patients on haemodialysis

**DOI:** 10.1186/s12879-022-07833-1

**Published:** 2022-11-14

**Authors:** Trodia Zitha, Chien-Yu Chen, Hatim Mudawi, Waleed Hussein, Maowia Mukhtar, Mazin Shigidi, Mohamed Elamin Awad Yousif, Mohammed Ahmed Ali, Dieter Glebe, Anna Kramvis

**Affiliations:** 1 Hepatitis Virus Diversity Research Unit, Department of Internal Medicine, School of Clinical Medicine, Faculty of Health Sciences, University of the Witwatersrand, 7 York Road, Parktown, 2193 Johannesburg South Africa; 2 Department of Medicine, Faculty of Medicine, University of Khartoum, Khartoum, Sudan; 3 Department of Immunology, Faculty of Medical Laboratory Sciences, Al-Neelain University, Khartoum, Sudan; 4 Department of Molecular Biology, Institute of Endemic Diseases, University of Khartoum, Khartoum, Sudan; 5 Dr. Salma Centre for Dialysis and Kidney Diseases and Transplantation, Khartoum, Sudan; 6 Department of Nephrology, Ibn Sina Specialized Hospital, Khartoum, Sudan; 7 Alnaw Teaching Hospital, Khartoum, Sudan; 8 Institute of Medical Virology, National Reference Centre of Hepatitis B viruses and Hepatitis D viruses, Justus Liebig University of Giessen, Giessen, Germany

**Keywords:** Hepatitis C virus, Genotype/subgenotype, End-stage renal disease, Haemodialysis, Nosocomial infection

## Abstract

**Background:**

Hepatitis C virus (HCV) is a global public health problem, with ~ 11 million people in Africa infected. There is incomplete information on HCV in Sudan, particularly in haemodialysis patients, who have a higher prevalence compared to the general population. Thus, our objectives were to genotype and molecularly characterize HCV isolated from end-stage renal disease haemodialysis patients.

**Methods:**

A total of 541 patients were recruited from eight haemodialysis centres in Khartoum and screened for anti-HCV. Viral loads were determined using in-house real-time PCR in seropositive patients. HCV was genotyped and subtyped using sequencing of amplicons of 5′ untranslated (UTR) and non-structural protein 5B (NS5B) regions, followed by phylogenetic analysis of corresponding sequences.

**Results:**

The HCV seroprevalence in the study was 17% (93/541), with HCV RNA-positive viremic rate of 7% (40/541). A low HCV load, with a mean of 2.85 × 10^4^ IU/ml and a range of 2.95 × 10^3^ to 4.78 × 10^6^ IU/ml, was detected. Phylogenetic analyses showed the presence of genotypes 1, 3, 4, and 5 with subtypes 1a, 1b, 1 g, 3a, 4a, 4 l, 4 m, 4 s, and 4t. Sequences of HCV from the same haemodialysis units, clustered in similar genotypes and subtypes intimating nosocomial infection.

**Conclusion:**

HCV infection is highly prevalent in haemodialysis patients from Sudan, with phylogenetic analysis intimating nosocomial infection. HCV genotyping is useful to locate potential transmission chains and to enable individualized treatment using highly effective direct-acting antivirals (DAAs).

## Introduction

Hepatitis C Virus (HCV) infects by blood-borne transmission, with transfusion of blood products and recreational intravenous drug injection being the main transmission routes [1]. Although proactive screening and strategized testing algorithms of transfusion-transmitted infections in high-income countries (HIC) have decreased HCV prevalence [2], screening using nucleic acid testing remains a major cost factor in low-middle-income countries (LMIC). HCV infection continues to be a global disease burden, with an estimated 58 million people living with chronic infection, 1.5 million newly infected annually, and 290 000 hepatitis C related annual deaths [3].

Chronic HCV infection may lead to the progression to end-stage liver diseases and eventual liver failure [1, 2]. Moreover, HCV has been associated with extra-hepatic manifestations [4] and complications including chronic renal disease [5]. Chronic HCV leads to increased risk of developing chronic kidney disease (CKD), a faster rate of renal function loss, higher incidence, accelerated progression from CKD to end-stage renal disease (ERSD), higher morbidity, and mortality [6–8].

HCV prevalence is < 1% in most HIC, being higher in LMICs [9]. In 2017, an ~ 11 million individuals were infected with HCV in Africa [10]. Patients on maintenance haemodialysis are at a higher risk of contracting HCV [5]. HCV prevalence amongst ESRD patients varies between HIC (3.4%) and LMIC (75–80%) [11]. Even with the global trend of decreased HCV prevalence in haemodialysis patients, the prevalence remains higher than in the healthy general population [12].

There is limited information on HCV prevalence and transmission in Sudan [13]. HCV prevalence in the general population ranges from 1.82 to 4.8% [14–17]. A few studies, conducted in the Sudanese haemodialysis population, show a higher prevalence ranging from 6 to 34.9% [18–21],

HCV is classified into eight phylogenetic clades designated as genotypes and 90 confirmed subtypes, with diverse geographic distribution [22–25]. Genotype 1 is the most prevalent genotype worldwide (49.1%), followed by genotypes 3 (17.9%), 4 (16.8%), 2 (11%), 5 (2%), and 6 (1.4%) [26]. Genotype 2 is common in the Mediterranean region, West Africa, and the Americas whereas genotype 3 is endemic in South America, South, and Central Asia. Genotype 4 is believed to have originated from Central Africa and to be prevalent there, in North Africa, the Middle East, and eastern sub-Saharan Africa. With 18 confirmed subtypes [22, 25], HCV genotype 4 has significant genetic divergence compared to other genotypes. Genotype 5 is mainly found in South Africa while genotype 6 predominates in South China and South-East Asia. Genotype 7 was identified in Central Africa, and genotype 8 was discovered in India [23, 27].

In Sudan, genotype 4 predominates (90%), followed by genotypes 1 (5%) and 2 (3%) [28]. Genotype 3 is detected from hepatitis patients in Khartoum State (2%) [29]. The known Sudanese HCV subtypes are 4c, 4d, and 4e [16]. Although, available data indicating the prevalence of genotypes 1, 2, 3, and 4 in the general Sudanese population, the information on their distribution in Sudanese haemodialysis population is still scarce. Updated comprehensive epidemiologic data are indispensable to implement strategies against HCV infection, including direct-acting antivirals (DAA) treatment for specific genotypes. The study aimed to determine the prevalence, genotype, and to molecularly characterize HCV isolated from Sudanese ESRD patients under maintenance haemodialysis.

## Material and methods

### Study design, recruitment and HBV/HCV prevalence

A prospective multi-centre cross-sectional hospital-based study was designed and conducted between December 2014 and January 2016. The power and sample size calculation was estimated using prevalence of ESRD in Sudan [30], known method with a 95% confidence interval [31]. In total, 541 haemodialysis patients were recruited from eight haemodialysis units in Khartoum, including Soba, Salma, Academy Hospital, Elturki, Ibn Sina, Tropical Hospital, Alnaw, and Police hospital. We included only individuals who were 18 years of age or older at the time data were collected. Information sheets and research concepts were given to research participants upon recruitment sessions. Informed consent for participation was signed and collected. Baseline demographic data and clinical profiles were recorded for all participants by study investigators. Socio-demographic data were obtained and recorded for all participants through a questionnaire including their age, gender, occupation, marital status, original and current residence areas. Clinical profiles documented included duration of dialysis in years, number of dialysis units attended, Hepatitis B Virus (HBV) vaccination and date, doses of HBV vaccination received, history of jaundice, history of dental procedure, intravenous drug usage, blood transfusions, frequency of blood transfusion, and diagnosis of chronic HCV.

Participant’s serum was collected by study investigators and screened for alanine aminotransferase (ALT) (hospital in-house serology testing), anti-HCV and hepatitis B surface Antigen (HBsAg) in Sudan using commercial captured ELISA methods as described by the manufacturer (Diasource, Nivelles, Belgium). The samples were transported in a temperature-controlled environment and stored in − 70 °C freezer upon arrival in South Africa.

### HBV DNA extraction

HBV DNA was extracted from serum using the QIAamp® DNA Blood Mini Kit (QIAGEN, GmbH, Hilden, Germany) following the manufacturer’s instructions. Best quality water (BQW) was included in the extraction as a negative control.

### Hepatitis B virus DNA detection

Hepatitis B virus was quantified using HBV-Taq1 and HBV-Taq2 primer sets together with the FAM/TAMRA labelled TaqMan BS-1 probe as previously described [32, 33]. The PCR reaction was performed using the Bio-Rad CFX96 real-time system (Bio-Rad Laboratories, Hercules, CA, USA). Serial dilutions of the plasmid encoding a single genome of HBV DNA ranging from 2 × 10^1^ to 2 × 10^11^ IU/ml were used to generate a linear standard curve. Second World Health Organization (WHO) International Standard for HBV Nucleic Acid Amplification Techniques (product code 97/750) from National Institute for Biological Standards and Controls (NIBSC; Hertfordshire, UK) was included as an internal positive control at the final concentration of 10^6^ IU/ml to calibrate and align the standard curve. Samples, serial dilutions of plasmid, and positive and negative controls were all tested in triplicate.

### Extraction and reverse transcription of RNA

HCV RNA was extracted from 140 μl serum using QIAamp® Viral RNA Mini Kit (Qiagen, GmbH, Hilden, Germany), with known HCV-negative, HCV-positive sera, and best quality water included as controls for extraction. Extracted HCV RNA was immediately processed to cDNA synthesis using Superscript® III first-strand synthesis system (Invitrogen, Carlsbad, CA, USA). An aliquot of 8 μl of HCV RNA was used per reaction to synthesize cDNA according to the manufacturer's instructions.

### Amplification of 5’UTR and NS5B regions, sequencing and phylogenetic analyses

Following cDNA synthesis, the 5’UTR region (genotyping) and NS5B region (subtyping) were amplified individually using nested polymerase chain reaction (PCR), using published primer sets [34, 35]. The NS5B region (subtyping) primers were modified to be subtype specific. HCVN5BG1/3-1F (nt 8262–8278) 5′-ACCCGYTGYTTTGACTC-3′; HCVN5BG1/3-1R (nt 8617–8634) 5′-TGGTCATAGCYTCCGTGA-3′; HCVN5BG1/3-3F (nt 8264–8278) 5′-CCGYTGYTTTGACTC-3′; HCVN5BG1/3-3R (nt 8616–8632) 5′-GTCATAGCY TCCGTGAA-3′; HCVN5BG4-1F (nt 8262–8278) 5′-ACCCGCTGYTTTG ACTC-3′; HCVN5BG4-1R (nt 8615–8632) 5′-CGTCATAGCYTCCGTGAA-3′; HCVN5BG4-3F (nt 8264–8278) 5′-CCGCTGYTTTGACTC-3′; HCVN5BG4-3R (nt 8616–8632) 5’GTCATAGCYTCCGTGAA-3′. Known HCV-negative, HCV-positive sera, best quality water, as well as full-length HCV pCV-H77 (genotype 1a), pCNJ4C6S (HCV genotype 1b), and pJ6CF2a (HCV genotype 2a) plasmid clones (obtained from Prof R. Purcell, NIH, USA) were included as controls.

The first-round PCR of 5’UTR region was carried out in a SimpliAmp Thermal Cycler (Thermo Fisher Scientific, Carlsbad, CA, USA), using Platinum SuperFi master mix (Invitrogen, Carlsbad, CA, USA) with 4 μl cDNA as template and 1 μM final concentration of each primer in a final volume of 25 μl PCR mix. The cycling conditions were: an initial activation at 95 °C for 45 s, followed by 40 cycles of denaturation at 98 °C for 17 s, annealing at 51 °C for 22 s, extension at 72 °C for 1 min 20 s, followed by a final extension at 72 °C for 5 min. The second-round PCR was carried out using the same master mix and amplification conditions as first-round PCR containing 5 μl of the first-round PCR product as a template, 1 μM final concentration of each primer in a final volume of 50 μl PCR mix. For the NS5B region, nested PCR amplification was carried out using the same master mix and conditions as described for 5′UTR.

Amplified PCR products were separated and detected by agarose gel electrophoresis. Positive results yielded a 251 bp amplicon for the 5’UTR region and a 368 bp amplicon for the NS5B region. Amplicons were prepared for sequencing using the BigDye™ Terminator v3.1 Cycle Sequencing Kit (Applied Biosystems, Foster City, USA). Sanger sequencing was performed by Inqaba Biotechnical Industries (Pty) Ltd (Pretoria, South Africa) using the ABI 350 XL genetic analyzer (Applied Biosystems, Foster City, CA, USA) with specific in house designed sequencing primers. The 5′UTR ad NS5B regions were analyzed in the forward direction of a single fragment. All sequences of HCV isolate have been deposited in GenBank.

Nucleotide sequences obtained in each region were aligned with HCV genotype and subtype reference sequences retrieved from GenBank using Molecular Evolutionary Genetics Analysis (MEGA) software package, version 7.0 [36]. Phylogenetic trees were further constructed by the maximum likelihood method using IQ-TREE 2 with GTR base substitution model [37]. The robustness of the tree topologies was estimated by bootstrap analysis with 1000 data sets, and only bootstrap values > 70 were displayed on the phylogram. Trees were visualized using the Interactive Tree Of Life (iTOL) v5 [38]. Statistical analysis was performed using the SPSS version 25.0 software (IBM, Armonk, North Castle, NY, USA). Student’s t-test was used to compare continuous variables. Fischer’s exact and Chi-square tests were used to determine the significant difference between categorical variables. A p-value of < 0.05 was considered statistically significant.

### HCV viral load determination

HCV viral load was quantified by in-house real-time PCR targeting the conserved 5’ UTR region. Briefly, the 25 μl reaction mix contained Taqman Fast Advanced master mix (Thermo Fisher Scientific, Carlsbad, CA, USA), 250 nM Taqman FAM/TAMRA probe HCVQ1TAQ (nt124-148) 5’-FAM-CCCTCCCGGGAGAGCCATAGTGGTC-TAMRA-3, 900 nM each of in-house designed forward HCVQ1F (nt74-91) 5’-AGCGTCTAGCCATGGCGT-3’ and reverse HCVQ1R (nt155-174) 5’-ATTCCGGTGTACTCACCGGT-3’ primers, and 2 μl each of known HCV-negative, HCV-positive sera, PCR grade water or HCV cDNA as template.

Complementary DNA (cDNA) was amplified in the Bio-Rad CFX96 real-time system (Bio-Rad Laboratories, Hercules, CA, USA) using the following cycling setting: an initial cycle at 50 °C for 2 min and 95 °C for 20 s followed by 40 cycles at 95 °C for 3 s and 60 °C for 30 s. Acrometrix™ HCV High Control (Thermo Fisher Scientific, Carlsbad, CA, USA) was included as a positive control. The real-time PCR was performed in duplicate. Serial dilutions of the plasmid DNA encoding full-length HCV genotype 1a genome (pCV-H77C) were used in triplicate to generate the standard curve. The dynamic range of HCV real-time PCR was estimated to be 1.86 × 10^3^–1.86 × 10^12^ IU/ml.

## Results

### HCV seroprevalence and RNA-positive (viraemic) rate in Sudanese ESRD patients

The HCV seroprevalence in the study was 17% (93/541). Of the 93 anti-HCV positive patients, 2 were HBsAg-positive, HBV/HCV co-infected. The remaining 91 HBsAg-negative samples of which 8 were found to be HBV DNA-positive and therefore hepatitis C with occult HBV infection (OBI). Amplification of the 5’ UTR and the NS5B region was performed on 93 anti-HCV-positive sera. A total of 62/93 samples were positive in the 5’UTR region and 42 in the NS5B region, with only 30.1% (28/93) of the samples being positive in both regions. The HCV RNA-positive viremic rate was 12.6% (68/541), PCR-positive in one or both regions.

### Genotyping of HCV using the 5′UTR region

**Fig. 1:**
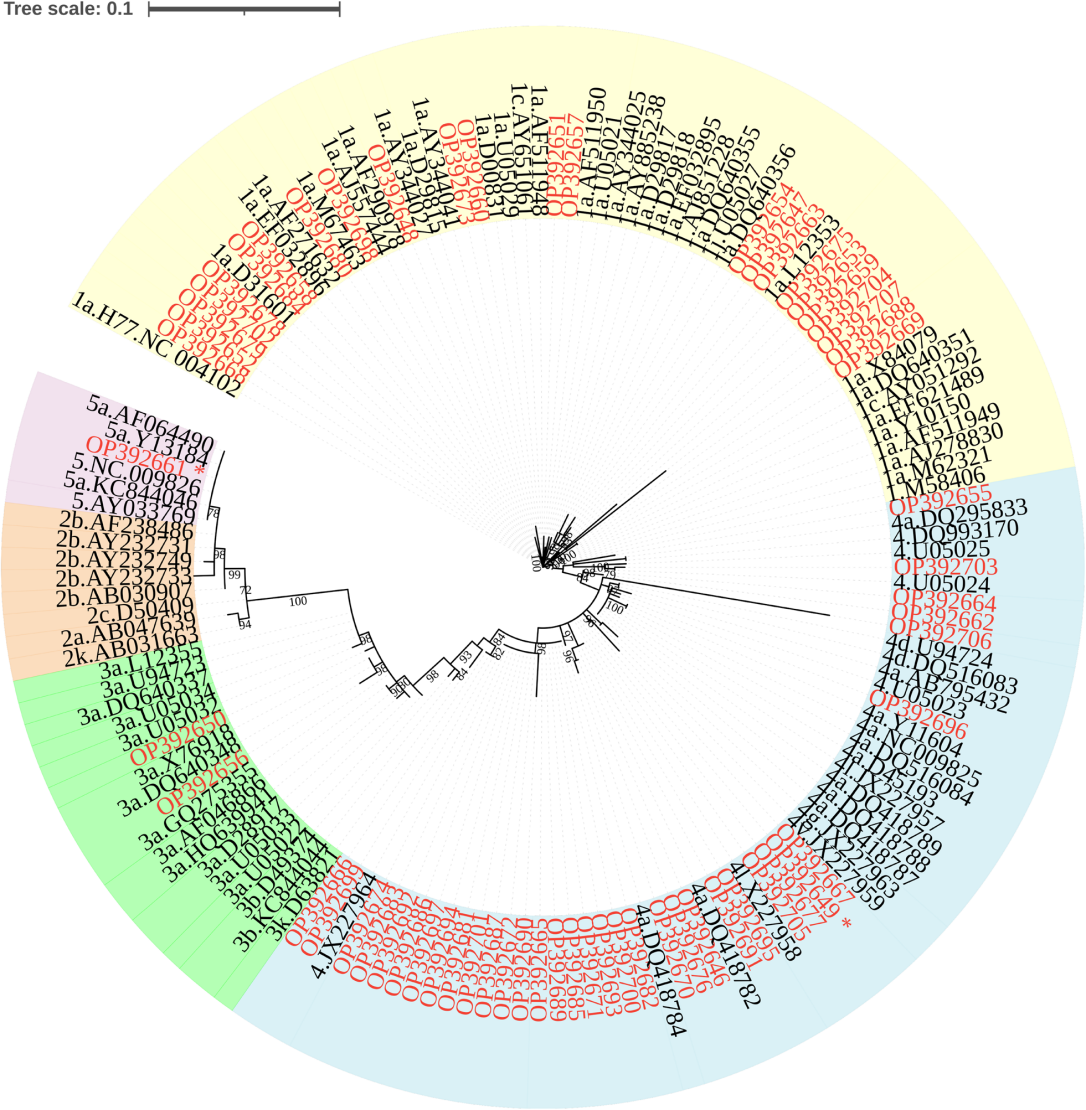
Phylogenetic analysis of the HCV 5’UTR region isolated from Sudanese end stage renal disease (ESRD) patients. Tree was constructed using maximum likelihood method in the IQ-TREE 2 software with GTR base substitution model and reference sequences from Genbank. Bootstrap statistical analysis was performed using 1000 replicates. The Sudanese isolates are shown in red. The discordant genotypes/subtypes of sequenced isolates are marked in asterisk

The PCR-positive samples were sequenced and subjected to phylogenetic analysis. Clustering of the sequences with reference sequences obtained from GenBank showed the presence of genotypes 1, 3, 4, and 5 (Fig. 1) among the Sudanese haemodialysis population. The genotype most frequently identified belonged to genotype 4 (56%). The remainder of the isolates included genotype 1 (39%), genotype 3 (3%), and genotype 5 (2%).

### Distribution of genotypes across dialysis centres

**Fig. 2:**
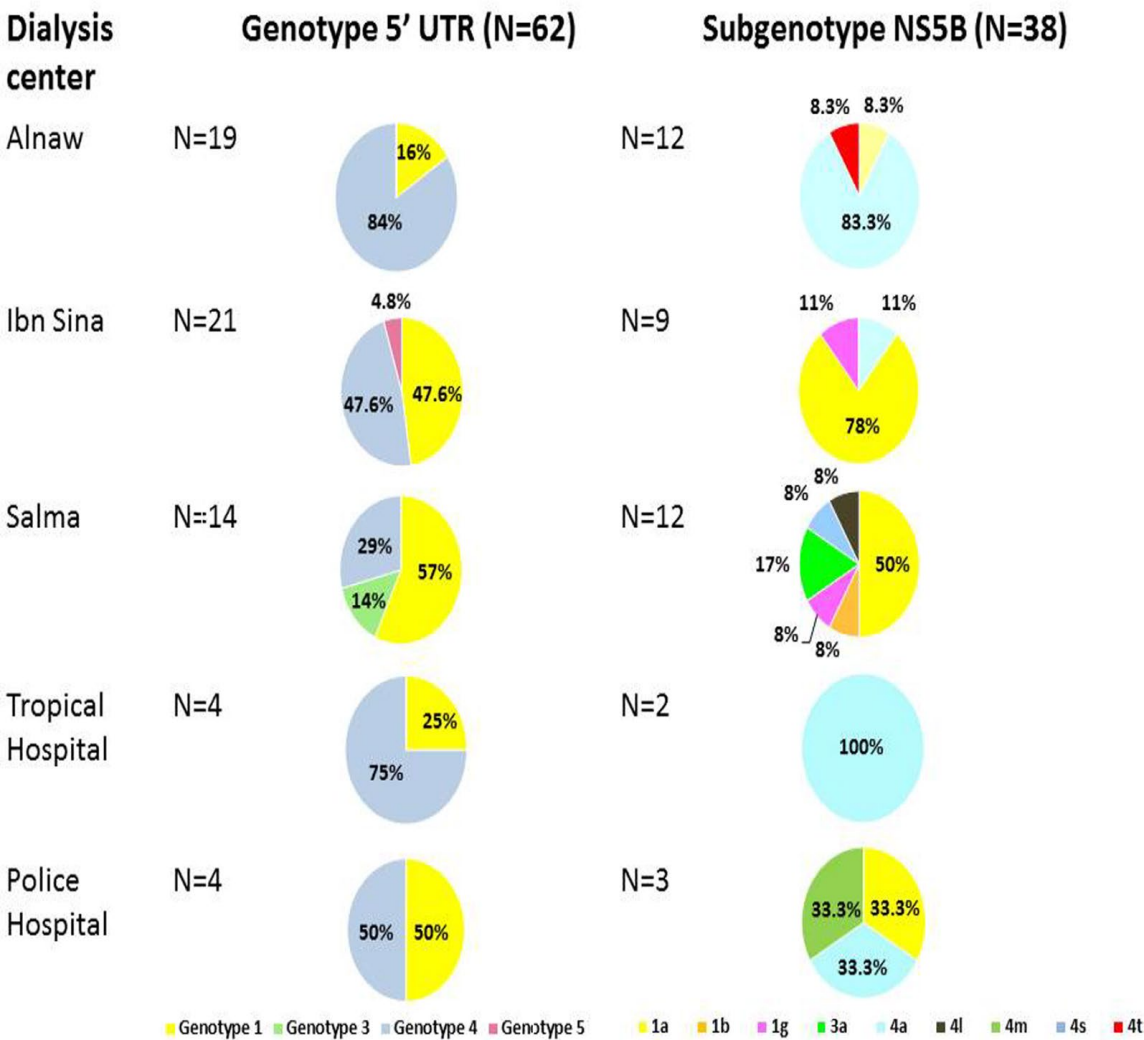
HCV genotype and subgenotype distribution among Sudanese haemodialysis centres. Specimen collected from Soba, Academy Hospital, and Elturki centres could not be successfully amplified to sequence the RNA. The discordant genotype/subtype isolates are included in this figure

HCV genotypes were successfully determined for HCV isolated from Alnaw, Ibn Sina, Police Hospital, Salma, and Tropical Hospital, using the phylogenetic analysis of the 5′UTR region sequences (Fig. 1) and their distribution across the five dialysis centres analysed (Fig. 2). Genotypes 1 and 4 were found in all centres. Dialysis centres Alnaw and Tropical Hospital had the highest prevalence of genotype 4. Genotype 5 was found in Ibn Sina, and genotypes 3 in Salma.

### The relationship between baseline characteristics of the participants and genotype distribution

Most of the HCV genotyping PCR positive participants were male N = 42 (76%) across all genotypes, had been undergoing haemodialysis for more than a year and were diagnosed with HCV after initiating haemodialysis. There was a statistically significant relationship between genotypes and the duration of haemodialysis treatment. Ninety-four percent (94%) of patients infected with genotype 4 had been on haemodialysis for less than a year, while most of the patients infected with genotype 1 being on haemodialysis for more than a year (96%). The likelihood ratio of the duration of haemodialysis treatment influencing the genotypes observed was found to be 13.668, p = 0.034. Phi and Crammer’s V tests were used to determine the level of association. Duration of haemodialysis has been observed to have a small to medium effect on influencing the possible genotypes. Another statistically significant relationship was observed between the genotypes and dialysis centres. The frequency of genotype 4 was statistically significantly higher (p < 0.05) in Alnaw (84%) and Tropical Hospital (75%) as compared to the other centres Police hospital (50%), Ibn Sina (47.6%), and Salma (29%). There was no statistically significant relationship between genotypes with gender (p = 0.721), age (p = 0.117), number of blood transfusions (p = 0.766), the diagnosis of chronic HCV (before or after dialysis) (p = 0.197), number of centres attended (p = 0.34), ALT levels (p = 0.165), and HCV viral loads (p = 0.201).

### Distribution of HCV subtypes in haemodialysis centres

**Fig. 3:**
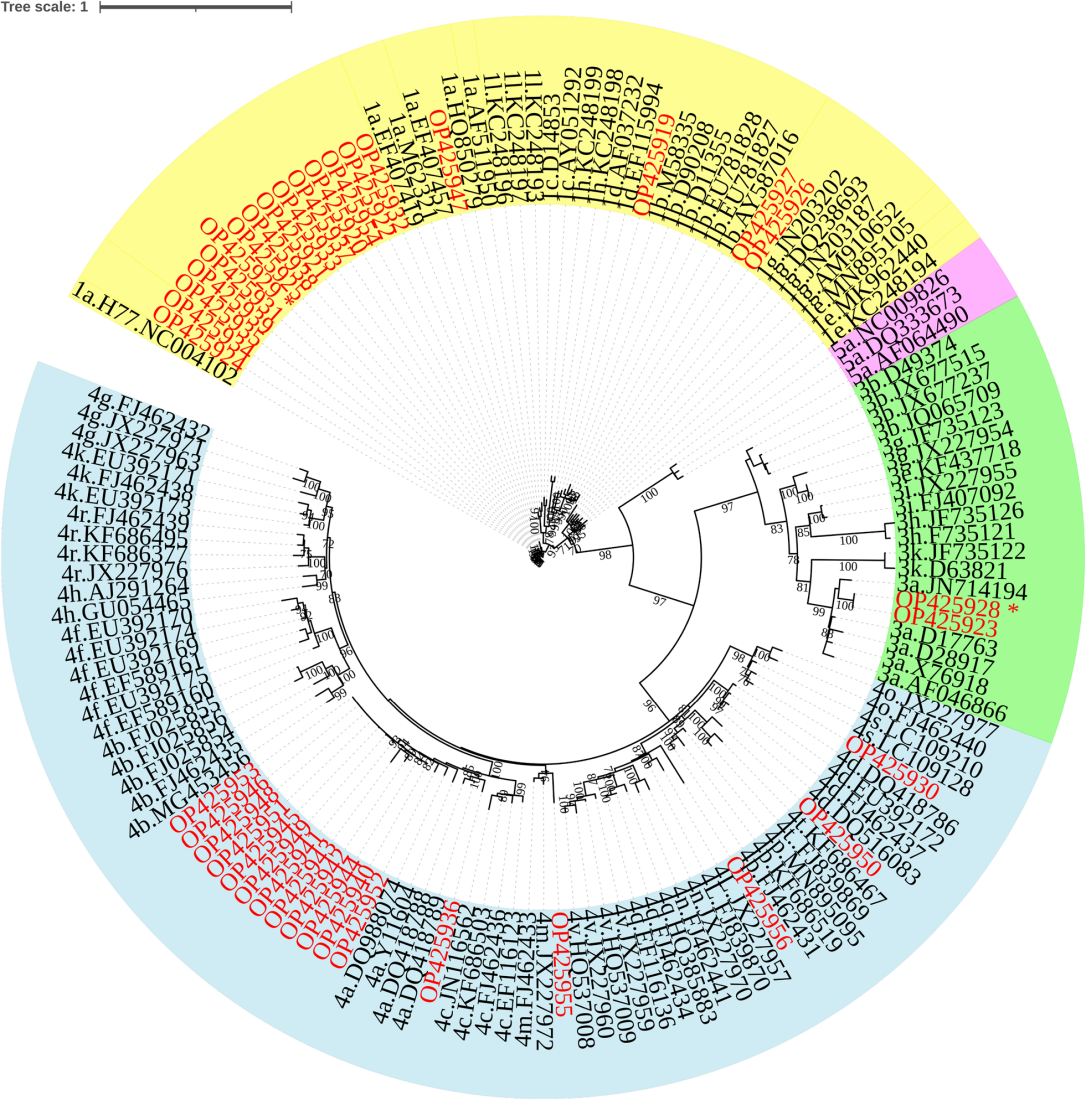
Phylogenetic analysis of the HCV NS5B region isolated from Sudanese end stage renal disease (ESRD) patients. Tree was constructed using maximum likelihood method in the IQ-TREE 2 software with GTR base substitution model and reference sequences from Genbank. Bootstrap statistical analysis was performed using 1000 replicates. The Sudanese isolates are shown in red. The discordant genotypes/subtypes of sequenced isolates are marked in asterisk

The genetically diverse NS5B region showed high variability in subtypes (Figs. 2 and 3). HCV subtypes detected included 1a, 1b, 1 g, 3a, 4a, 4 l, 4 m, 4 s, and 4t. Most HCV isolates belonged to subtype 1a (39.5%) followed by subtype 4a (36.8%). Subtypes 1b (2.6%), 1 g (5.3%), 3a (5.3%), 4 l (2.6%), 4 s (2.6%), 4 m (2.6%) and 4t (2.6%), were found at a lower frequency compared to subtypes 1a, 4a. Two samples were discordant in genotype (OP3392649 and OP392661) (Fig. 1) and subtype classifications (OP425928 and OP425929) (Fig. 3).

### Quantification of HCV using real-time PCR

Viral loads could be determined using real-time PCR for 66% of the samples tested (61/93). However, the viral loads for 24/61 fell below the assay’s detection limit and were excluded from further analysis. A total of 37/93 (40%) samples had HCV viral loads ranging from 2.95 × 10^3^–4.78 × 10^6^ IU/ml with the mean HCV viral load of 2.85 × 10^4^ IU/ml. Mean viral loads between dialysis centres were found to be 3.71 × 10^4^ IU/ml in Salma, 2.75 × 10^4^ IU/ml in Alnaw, 2.63 × 10^4^ IU/ml in Ibn Sina, 3.02 × 10^4^ IU/ml in Tropical Hospital and 1.48 × 10^4^ IU/ml in Police hospital. When viral loads between genotypes were analysed, genotype 1 samples had a mean viral load of 2.95 × 10^4^ IU/ml while genotype 4 samples were found with a mean viral load of 2.63 × 10^4^ IU/ml, p > 0.05.

There was no statistically significant relationship between viral loads and the dialysis centres attended (p = 0.466), age (p = 0.643), gender (p = 0.648), time on haemodialysis (p = 0.405), when chronic HCV was diagnosed (p = 0.705), and ALT levels (p = 0.594).

Higher viral loads were associated with receiving haemodialysis treatment from more than one centre while lower viral loads were observed in patients who only attended one centre. A statistically significant relationship (p < 0.05) was observed between the number of blood transfusions received and HCV viral loads (p = 0.006). Lower viral loads were observed in patients who have had more than one blood transfusion as compared to patients who received blood transfusion only once.

## Discussion and conclusion

Despite good clinical practices employed in dialysis centres to prevent nosocomial infections, the prevalence of HCV in these centres continues to be high. Few studies have been conducted to determine the prevalence of HCV in haemodialysis populations in Sudan.

In the present study, 17% of 541 patients were reactive for anti-HCV, within the range reported for haemodialysis patients [18, 20, 21, 29], and comparable with prevalence of 15.2% in Sudanese haemodialysis patients [29]. Approximately 1.5% of the study population had HCV with OBI. OBI is defined as the presence of HBV DNA in serum in the absence of HBsAg [39]. OBI in HCV-infected patients can play a role in accelerating the development of cirrhosis and hepatocellular carcinoma [40].

In this study, different amplification frequencies between the 5’UTR and NS5B region were noted: higher in the 5’UTR than the NS5B, in agreement with published data [41, 42]. The highly conserved nature of the 5’UTR compared to the more variable NS5B could account for the difference [43]. Furthermore, NS5B is closer to the 3’ HCV RNA, which is more prone to RNA degradation. Although the NS5B is a relatively difficult region to amplify, it enabled the HCV subtype determination.

Overall, 26/28 of the sequences (92.8%) were concordant in the NS5B and 5’UTR regions. Phylogenetic analysis of the two regions identified the presence of genotypes 1, 3, 4, and 5. Similarly, genotypes 1, 2, 3, and 4 were detected in the haemodialysis population in the Middle East and North Africa [44]. HCV genotype 4 predominates in the Sudanese general population [15], As expected, the presence of genotype 3, which circulates in Sudan, Egypt, and some parts of Central Africa [29, 45], was found in lower frequency [29]. The high prevalence of genotype 1 agrees with its predominance in dialysis patients in other regions of the world [46, 47]. Genotype 5a, which predominates in South Africa [48], was detected in the present study. Several possibilities can explain the presence of this genotype. Firstly, the introduction of genotype 5a in the Sudanese population may have been brought about by infected travellers coming from South Africa to Sudan. Secondly, the genotype observed as genotype 5a may have been misclassified as subgenotypes 1a and 5a have 96.2%–96.5% similarity and the NS5B region of the isolate clustered with subgenotype1a. Lastly, it is possible following coinfection/superinfection, this was a recombination event, albeit rare in vivo [49, 50] and difficult to detect [51]. Recombination was previously identified by sequencing the 5’UTR and NS5B regions [52, 53]. Furthermore, discordant genotype 4 (5’UTR)/subtype 3a (NS5B) was also detected in the cohort. The reason for the discordance of the two isolates was not determined in the present study. These putative recombinants would require confirmation by full genome molecular characterization to identify the recombination points. In addition to the known subtypes in Sudan (4c/d/e) [16], subtyping detected subtypes 1a, 1b, 1 g, 3a, 4a, 4 l, 4 m, 4 s, and 4t. Despite subtype 1 g was previously found in Europe, Canada and Africa [54], it was isolated from participants in the Netherlands and Germany with Sudanese origin [55, 56].

Several risk factors associated with HCV transmission in dialysis centres exist. These factors include time on dialysis [57], dialysis in multiple centres [46], blood transfusion [58], patients undergoing dialysis on the same shift, and poor infection control practices [59]. The current analysis displayed that majority of the patients may have contracted HCV after starting haemodialysis. None of the HCV-positive patients had a history of intravenous drug abuse and other modes of transmission were not implicated. There was a significant relationship between the dialysis centre attended and genotypes present. Genotypes 1 and 4 were detected in all the haemodialysis centres. The distributions of genotypes in dialysis centres were uneven except Ibn Sina and Police Hospital had equal distribution of the two genotypes. Salma had more genotype 1 variants while genotype 4 prevailed in dialysis centres Alnaw and Tropical Medicine. This uneven distribution of genotypes in the dialysis centres intimates nosocomial infection. Previous studies have demonstrated that detecting the presence of the virus using HCV antibody and liver enzymes may produce negative results in the presence of the virus [60–62]. Thus, this is a possible explanation for not detecting HCV infection at the outset of treatment. The number of years spent on haemodialysis is a risk factor for HCV transmission [57], with patients on longer duration on haemodialysis treatment having a high risk of contracting HCV compared to patients with a shorter duration [60]. Thus regular, routine nucleic acid testing, which differentiates active from inactive HCV infection, is recommended. The correlation between genotype and duration of haemodialysis observed can also result from the shift in the genotype prevalence from 2014 onwards. Genotype 4 has become the dominate genotype in Sudanese chronic HCV (92%) [63], haemodialysis and general populations (92%) [64].

In agreement with previous studies [65, 66], low viral loads were detected in Sudanese haemodialysis patients. Viral load is known to decrease significantly during the dialysis session but to return to the base level before the next dialysis [67]. This observation can be explained by the fact that HCV is larger than the dialysis membrane pore thus virions are adsorbed onto the inner surface of the filter membrane during dialysis [68]. Although not proved experimentally, it has been suggested that adsorbed virions could possibly be destroyed by the hydraulic pressure utilized during dialysis [69]. Although this may be the case, the dialysis treatment does not clear the virus but can result in decreased HCV RNA levels detected [67, 70]. There was no significant difference between HCV viral load and ALT levels. Our results were in agreement with that of others in that the ALT level is not a predictive value for HCV infection [70, 71].

There are a few limitations in the current study: 24 patients had HCV cDNA detected with the value below the lower quantification limit. This could be due to the in-house real-time PCR assay was not sensitive enough to detect the viral loads below 1.86 × 10^3^ IU/ml and poor storage and handling of samples during specimen collection and/or transportation may have contributed to RNA degradation.

The higher seroprevalence of HCV in the haemodialysis patients compared to the general population, the uneven genotype distribution in the different dialysis centres, and the genotype difference in patients with shorter haemodialysis duration (genotype 4) compared to longer duration (genotype 1), implicate nosocomial infection. Moreover, the two samples, which were infected with subtype 3a have 100% nucleotide sequence identity in the NS5B fragment, and had a high (100) bootstrap support (Fig. 3). These two patients were attending haemodialysis treatment at the same centre and thus this may be a sign of a nosocomial infection. Since there is no vaccine available for HCV, stringent measures are necessary to prevent HCV transmission during haemodialysis. Before starting haemodialysis, all patients should be tested for HCV and regular testing should be done thereafter. HCV-positive patients should be treated with DAAs and dialysis performed in dedicated areas. Genotyping/subtyping of HCV is useful in tracing nosocomial infections and transmission chains in dialysis centres.

## Data Availability

All the information supporting our conclusions and relevant references are included in the manuscript. The dataset used and/or analysed during the current study is available in the GenBank repository. https://www.ncbi.nlm.nih.gov/genbank/. GenBank accession numbers for the 5’UTR sequences are OP392646, OP392647, OP392648, OP392649, OP392650, OP392651, OP392652, OP392653, OP392654, OP392655, OP392656, OP392657, OP392658, OP392659, OP392660, OP392661, OP392662,OP392663, OP392664, OP392665, OP392666, OP392667, OP392668, OP392669, OP392670, OP392671, OP392672, OP392673, OP392706, OP392707, OP392674, OP392675, OP392676, OP392677, OP392678, OP392679, OP392680, OP392681, OP392682, OP392683, OP392684, OP392685, OP392686, OP392687, OP392688, OP392689, OP392690, OP392691, OP392692, OP392693, OP392694, OP392695, OP392696, OP392697, OP392698, OP392699, OP392700, OP392701, OP392702, OP392703, OP392704, and OP392705. GenBank accession numbers for the NS5B sequences are OP425919, OP425920, OP425921, OP425922, OP425923, OP425924, OP425925, OP425926, OP425927, OP425928, OP425929, OP425930, OP425931, OP425932, OP425933, OP425934, OP425935, OP425936, OP425937, OP425938, OP425939, OP425940, OP425941, OP425942, OP425943, OP425944, OP425945, OP425946, OP425947, OP425948, OP425949, OP425950, OP425951, OP425952, OP425953, OP425954, OP425955, and OP425956.

## Abbreviations

ALT: : Alanine aminotransferase
DAAs: : Direct-acting antivirals
ERSD: : End-stage renal disease
HBV: : Hepatitis B Virus
HCV: : Hepatitis C virus
HIC: : High-income countries
iTOL: : Interactive tree of life
LMIC: : Low-middle-income countries
MEGA: : Molecular evolutionary genetics analysis
NS5B: : Non-structural protein 5B
UTR: : Untranslated region
